# One-Year Nutritional and Metabolic Changes in Patients on Incremental Peritoneal Dialysis Under Different Protein Diets: A Multicenter Pragmatic Cohort Study

**DOI:** 10.3390/nu18152565

**Published:** 2026-08-06

**Authors:** Silvio Borrelli, Carlo Garofalo, Tino Paolo Ambrosino, Vincenzo Bellizzi, Simona Andriella, Federica Marzano, Roberta Germanò, Chiara Ruotolo, Gianfranca Cabiddu, Roberto Russo, Valerio Vizzardi, Diana Bertoni, Adamasco Cupisti, Loris Neri, Gaetano Alfano, Gennaro Argentino, Silvia D’Alonzo, Paolo Chiodini, Giuseppe Paribello, Francesco Trepiccione, Roberto Minutolo, Luca De Nicola, I-COPE Group

**Affiliations:** 1 Unit of Nephrology, Department of Advanced Medical and Surgical Sciences, University of Campania “Luigi Vanvitelli”, 81031 Naples, Italy; carlo.garofalo@unicampania.it (C.G.); tinopaolo.ambro@gmail.com (T.P.A.); simo.andriella@gmail.com (S.A.); fedemarzy@gmail.com (F.M.); robertagermano84@gmail.com (R.G.); chiara.ruotolo@yahoo.it (C.R.); roberto.minutolo@unicampania.it (R.M.); luca.denicola@unicampania.it (L.D.N.); 2 “Sant’Anna e San Sebastiano” Hospital, 81100 Caserta, Italy; vincenzo@bellizzi.eu; 3 Unit of Nephrology and Dialysis, University of Cagliari, 09121 Cagliari, Italy; gianfrancas.cabiddu@unica.it; 4 Unit of Nephrology and Dialysis, University of Bari, 70124 Bari, Italy; roberto.russo@policlinico.ba.it; 5 Unit of Nephrology and Dialysis, Azienda Socio-Sanitaria Territoriale -Spedali Civili di Brescia, 25123 Brescia, Italy; vvizzardi@aslto4.piemonte.it (V.V.); diana.bertoni@asst-spedalicivili.it (D.B.); 6 Unit of Nephrology and Dialysis, University of Pisa, 56127 Pisa, Italy; adamasco.cupisti@med.unipi.it; 7 Unit of Nephrology and Dialysis, Azienda Sanitaria Locale CN2, 12051 Alba, Italy; lorisneri1960@gmail.com; 8 Unit of Nephrology and Dialysis, Azienda Ospedaliera di Modena, 41125 Modena, Italy; gaetano.alfano@unimore.it; 9 Unit of Nephrology and Dialysis, Ospedale del Mare, 80147 Napoli, Italy; gennaro.argentino@aslnapoli1centro.it; 10 Unit of Nephrology and Dialysis, Policlinico Gemelli, 00168 Roma, Italy; silvia.dalonzo@policlinicogemelli.it; 11 Medical Statistics Unit, University of Campania “Luigi Vanvitelli”, 81031 Naples, Italy; paolo.chiodini@unicampania.it; 12 Division of Nephrology, University Federico II, 80126 Naples, Italy; peppepary@hotmail.it; 13 Unit of Nephrology and Dialysis, Department of Medical Translational Sciences, University of Campania “Luigi Vanvitelli”, 81031 Naples, Italy; francesco.trepiccione@unicampania.it; 14 Biogem, Institute of Molecular Biology and Genetics, 83031 Ariano Irpino, Italy

**Keywords:** incremental peritoneal dialysis, low-protein diet, protein intake

## Abstract

**Background**: Commencing peritoneal dialysis (PD) with a lower dose (incremental PD) is a common strategy to improve patient acceptance and reduce treatment burden. However, specific dietary protein recommendations for incremental PD are lacking. **Methods**: An interim analysis of the I-COPE PD study cohort aimed to assess one-year changes in serum albumin, body mass index (BMI), and metabolic parameters in a cohort of patients on incremental PD. Following local protocols, patients were categorized into two groups according to protein diet prescription: protein diet (PrD) ≤ 0.6 and PrD > 0.6 g/kg/day. Longitudinal changes at baseline, 6 and 12 months were analyzed using mixed-effects models adjusted for age, sex, diabetes, residual kidney function and serum albumin at baseline. **Results**: We included 205 patients (mean age 63.3 ± 14.2 years; 66% male; 30% diabetes; 92.2% CAPD) and in 45.4% a PrD ≤ 0.6 g/kg/day was prescribed. Baseline demographic, clinical, lab and therapeutic characteristics were well-balanced between the two diet groups. After one year of follow-up, the prevalence of patients persisting in incremental PD was not different between the two dietary groups (59.1% in PrD ≤ 0.6 vs. 63.3% in PrD > 0.6, log-rank test = 0.759), and both groups showed a similar trend in serum albumin, characterized by an initial decline followed by stabilization (*p* = 0.647), with no significant differences in BMI (*p* = 0.461). Despite comparable dialysis adequacy, patients on PrD ≤ 0.6 exhibited significantly lower serum urea, phosphate, and potassium levels over time compared to the PrD > 0.6 group. **Conclusions**: In patients starting incremental PD, the prescription of a lower protein diet could be associated with better control of uremic toxins, while serum albumin levels over time remain comparable to those observed in patients under a higher-protein diet.

## 1. Introduction

Peritoneal dialysis (PD) is a home dialysis that is underutilized unless it demonstrates benefits in efficacy and cost saving compared to hemodialysis [1]. Incremental PD, in which patients start dialysis with a lower dose than the standard schedule, may improve acceptance of dialysis burden and its adoption has been growing in recent years [2]. The decision to adopt an incremental approach primarily hinges on having sufficient residual renal kidney function (RKF > 5 mL/min), which ensures a baseline level of dialysis adequacy by taking into account both renal function and dialysis treatment itself.

Incremental PD has demonstrated comparable patient survival during the first year of treatment when compared to standard dialysis [3,4,5]. Moreover, reducing PD exchanges may offer additional benefits, such as reducing exposure of peritoneum to the toxic effects of glucose, lowering the risk of peritonitis, and improving overall QOL [6,7]. Interestingly, this approach aligns with the recent international PD guidelines that prioritize the well-being and quality of life (QOL) of individuals undergoing treatment [2].

Patients on incremental PD face a delicate trade-off between dietary protein intake (DPI) and dialysis adequacy. High dietary protein intake may exceed the removal capacity of an incremental schedule, forcing an early dose increase. Conversely, low dietary protein intake may increase sarcopenia risk, exacerbated by peritoneal protein and amino acids losses [8]. Consequently, the nutritional management of these patients remains a critical, though overlooked, area of research.

Current guidelines generally recommend restricting dietary protein intake (0.6–0.8 g/kg of Body Weight/day) for stable patients with not-dialysis chronic kidney disease (ND-CKD), while suggesting higher intake for those on standard PD to compensate for membrane losses (>1.2 g/kg of Body Weight/day) [9,10].

No specific recommendation on protein diet is currently available for incremental dialysis. This knowledge gap often forces nephrologists to make individual prescribing decisions without clear evidence regarding the effects of different protein regimens.

We conducted an interim analysis of a cohort from the I-COPE PD study including patients starting dialysis on incremental PD. We compared the 12-month changes in nutritional, clinical, and metabolic parameters in patients under ≤ or >0.6 g/kg of BW/day.

## 2. Materials and Methods

This pre-specified analysis of the I-COPE CKD study evaluates one-year changes in nutritional (serum albumin and BMI), and metabolic parameters (serum urea, potassium, phosphate, and hemoglobin) treated with incremental PD stratified by dietary prescription (protein diet, PrD≤ vs. >0.6 g/kg/day).

Briefly, the I-COPE CKD study is a non-profit, observational study conducted across 20 Italian PD units, aimed at assessing the time to full dose in patients requiring renal replacement therapy (RRT). The study compared the time to full dose between two groups under medical therapy in nephrology: patients adding a low dose of PD (incremental) vs. patients continuing medical care because of refusal of any RRT. Starting dialysis was recommended if patients reached CKD stage 5 and had at least two complications that were not responsive to medical therapy (diet and drugs). The combined primary endpoint of I-COPE, that is, time to full dose, was defined as the interval between baseline (need of starting RRT) and the need of switching to a full-dose regimen (>2 daily CAPD dwells or >4 weekly APD sessions) to achieve a weekly Kt/V > 1.7, or until transition to hemodialysis, transplantation, or death [11].

In this analysis, we included only patients initiating incremental PD (n = 205). Exclusion criteria were refusal of low-dose PD, residual kidney function (RKF) < 5 mL/min/1.73 m^2^, advanced heart failure, severe cirrhosis, or a life expectancy of less than six months.

### 2.1. Data Collection

Baseline data included serum and urinary data, and pharmacological therapies. Laboratory and clinical parameters were recorded at each follow-up visit (6 and 12 months). Blood pressure (BP) was measured with an oscillometric sphygmomanometer according to the international hypertension guidelines and averaged from three readings taken at 5 min intervals [12]. In addition to clinical assessments for overhydration at each visit, patients kept home diaries to report body weight (BW) and home BP measurements. The estimated glomerular filtration rate (eGFR) was calculated using the Chronic Kidney Disease Epidemiology Collaboration (CKD-EPI) equation [13].

### 2.2. Variables of Exposure

Given the lack of established nutritional guidelines for protein intake in patients initiating incremental PD, dietary prescriptions followed local center protocols. The I-COPE-CKD group on incremental PD was categorized into two groups based on the protein diet prescription of each PD unit: higher than 0.6 g/kg/day and lower than 0.6 g/kg/day. The 0.6 g/kg/day threshold is widely recognized in the nephrology community as the standard definition for a “Low-Protein Diet” in the management of CKD stages 4–5, as supported by international guidelines [9,10]. In the context of incremental PD, this cutoff was chosen to assess how nephrologists managed the transition from conservative to conservative and low-dose dialysis.

Dietary protein intake was assessed using the Maroni formula, based on 24 h urinary urea nitrogen excretion [14] and ideal BW according to the Devine formula [9]. Both diets provided 30–35 kcal/kg/day, with controlled intakes of phosphate (600–800 mg/day), potassium (40–60 mmol/day), and sodium (<100 mmol/day). Fluid restriction was also recommended [9,10]. Dietary plans were further tailored to accommodate individual patient preferences. These regimens were prescribed at least three months prior to baseline (start of PD) and maintained thereafter based on clinical judgment.

### 2.3. Outcomes

The primary outcome of the current analysis was to evaluate changes in nutritional status (serum albumin, BMI) and metabolic (serum urea, phosphate, hemoglobin, potassium) parameters over the first year of incremental PD stratified by protein diets (PrD≤ or >0.6 g per kg of BW a day).

### 2.4. Statistical Analysis

Continuous variables are reported as mean (± standard deviation) or median (interquartile range), according to their distribution, which was assessed using the Shapiro–Wilk test. Between-group comparisons were performed using the unpaired *t*-test or the Wilcoxon–Mann–Whitney test, as appropriate. Categorical variables are presented as percentages and compared using the chi-square test or Fisher’s exact test.

Survival analysis was conducted to evaluate the probability of maintaining the incremental PD regimen. Persistence on incremental PD was estimated using the Kaplan–Meier method, and differences between groups were evaluated with the log-rank test. Dropout was defined as a transition to a full-dose regimen (>2 daily CAPD dwells or >4 weekly APD sessions), transfer to hemodialysis, transplantation, or death.

Longitudinal changes during the 12-month follow-up (at baseline, 6, and 12 months) were evaluated using linear and generalized linear mixed-effects models for continuous and categorical variables, respectively. The data structure consisted of three nested levels: repeated measurements over time (Level 1) nested within individual patients (Level 2), who were further nested within clinical centers (Level 3). The fixed-effects component of the model included the primary predictor of interest, PrD, time (baseline, visit at 6 and 12 months), and their two-way interaction term (time × PrD) to evaluate whether changes in variables of interest over time differed significantly between dietary groups. The model was adjusted for the following baseline and time-varying covariates to control for potential confounding: age, sex, diabetes status, baseline RKF and serum albumin. To account for the non-independence of observations, the model incorporated two hierarchical random intercepts (patient and center). Parameter estimation was performed using Restricted Maximum Likelihood (REML) to obtain unbiased variance components. Following model estimation, a comprehensive post-estimation analytical framework was applied to assess the overall global significance of differing longitudinal trajectories and predictive marginal linear predictions for each group at each specific time point. Finally, unadjusted pairwise comparisons with marginal linear effects were conducted to test both the differences between dietary groups within each time point and the longitudinal evolution within each group. Longitudinal changes in binary outcomes over time were evaluated using Generalized Estimating Equations (GEE) models. This approach was selected for its robustness in handling unbalanced datasets and missing observations due to incremental PD dropouts, which are inherent to repeated-measures designs [15].

Statistical analyses were performed using Stata software [Stata v. 14.2 (StataCorp, College Station, TX, USA)].

## 3. Results

Two hundred and five patients commencing low dose PD were included in this analysis. Main demographic features of the study cohort are listed in Table 1. Briefly, patients aged 63.3 ± 14.2 years, two-thirds were males, 30% had diabetes, and a major part of them (92.2%) started dialysis by CAPD.

Of the prescribed protein diets, 45.4% were on PrD ≤ 0.6 g/kg/day.

There was no difference in the distribution of the age, sex, CVD and diabetes, and clinical features between the two diet groups. The eGFR at dialysis start was similar in the two diet groups (*p* = 0.958) and no significant difference evident in serum levels of albumin, hemoglobin, calcium, phosphate, PTH and C reactive protein, sodium, and potassium were found. Notably, serum urea was significantly higher in PrD > 0.6 compared to PrD ≤ 0.6 g/kg/day (Table 1).

At baseline, the actual protein intake in PrD ≤ 0.6 group was effectively lower than patients on PrD > 0.6.

No difference in type of PD was found, since the use of CAPD was similar in the two diet groups; however, at baseline, the percentage of patients using one dwell a day was significantly higher in PrD < 0.6 (64%) than PrD > 0.6 group (44%; *p* = 0.008). No difference in icodextrin use was found. At baseline, PrD ≤ 0.6 was associated with a significantly lower number of anti-hypertensive drugs compared to PrD > 0.6 patients (−0.34 [−0.01; −0.67]) with no difference in the types of anti-hypertensives. Similarly, there was no difference in the prescription of statins, phosphate binders, calcium supplements and potassium exchangers between two groups. Moreover, a higher prescription of cholecalciferol in PrD ≤ 0.6 group was found, while no difference in calcitriol and paricalcitol was registered. The prescription of epoetin was lower in PrD ≤ 0.6 group and, similarly, the epoetin dose was significantly lower in PrD ≤ 0.6 group compared to PrD > 0.6 group (Table 2).

### Longitudinal Changes

**Figure 1:**
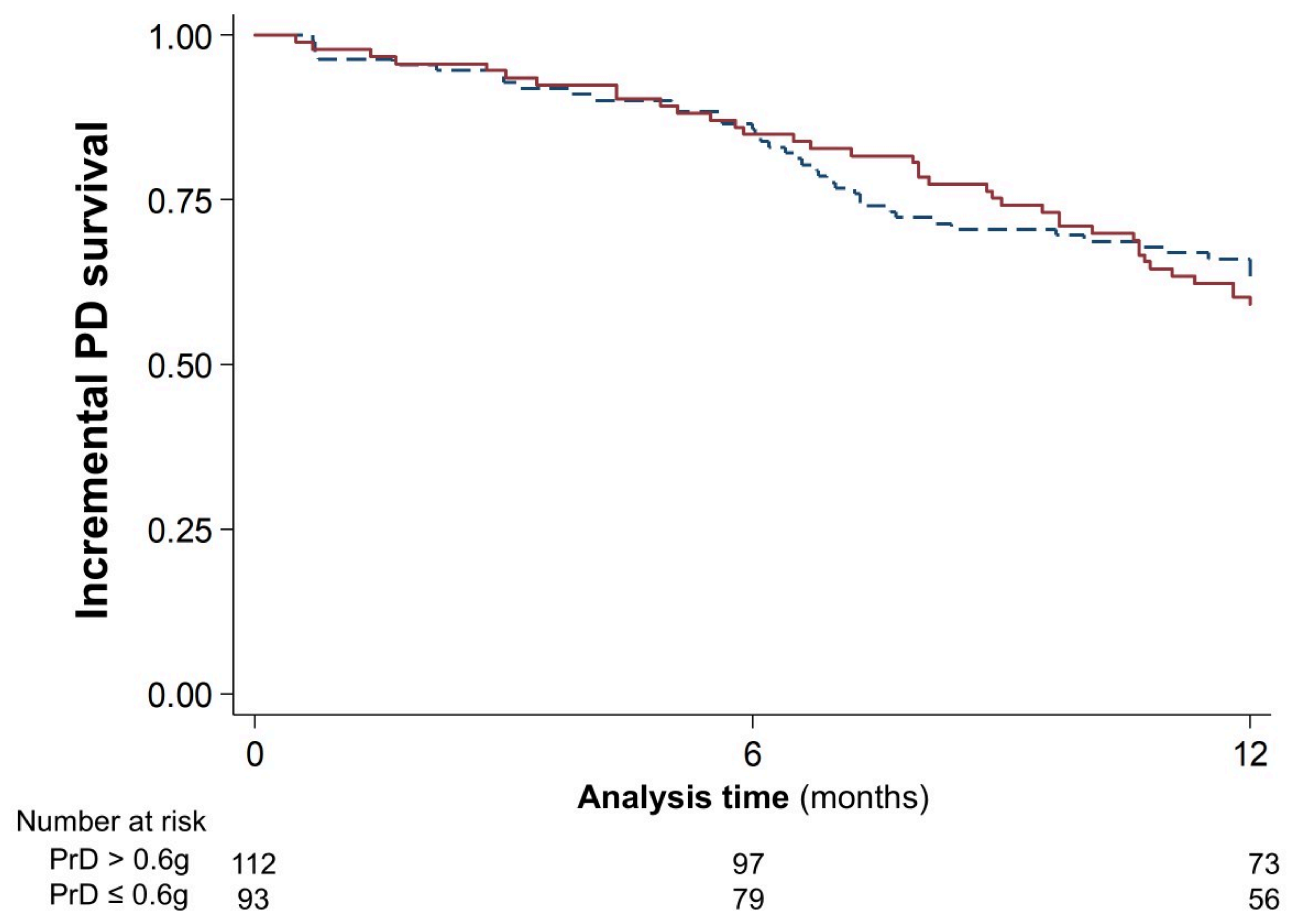
Kaplan–Meier analysis to evaluate the probability of incremental PD survival in ≤0.6 g/kg BW/day (solid maroon line) and >0.6 g/kg BW/day (dashed navy line).

After one year of follow-up, the percentage of patients persisting in incremental PD was 63.3% [95% CI: 53.7–71.5] in PrD ≥ 0.6 and 59.1% in PrD < 0.6 [95% CI: 48.5–68.3]. As described in Figure 1, no statistical difference in the probability of incremental PD survival associated with <0.6 was observed (*p* = 0.759). The 12-month prevalence of different component of composite outcome was similar in the two groups: full-PD 12.7% [95% CI: 8.5–18.0] in PrD ≤ 0.6 vs. 13.7% [95% CI: 9.3–19.1] in PrD > 0.6; HD 2.0% [95% CI: 0.5–4.9] in PrD ≤ 0.6 vs. 2.4% [95% CI: 0.8–5.6] in PrD > 0.6; Kidney Transplantation 2.4% [95% CI: 0.8–5.6] in PrD ≤ 0.6 vs. 2.4% [95% CI: 0.8–5.6] in PrD > 0.6; and all-cause death 1.0% [95% C.I.: 0.1–3.5] in PrD ≤ 0.6 vs. 2.5% [95% CI: 0.8–5.6] in PrD > 0.6.

**Figure 2:**
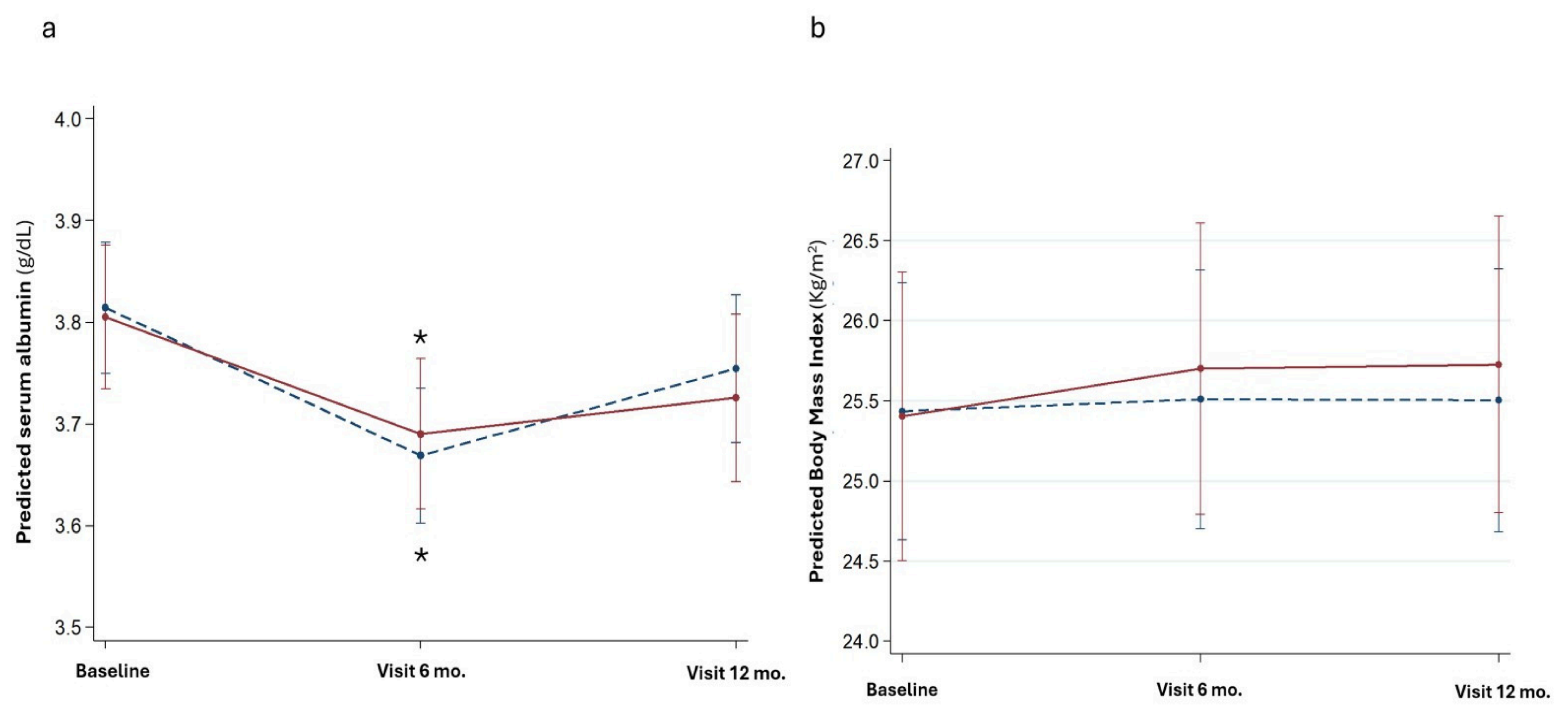
One-year changes in serum albumin (panel (**a**)) and Body Mass Index (panel (**b**)) in ≤0.6 g/kg BW/day (solid maroon line) and >0.6 g/kg BW/day (dashed navy line). * *p* < 0.05 for intragroup comparisons.

A linear mixed-effects regression showed a reduction in serum albumin over time in whole cohort, decreasing from 3.82 g/dL (95% CI: 3.76–3.87) at baseline to 3.69 g/dL (95% CI: 3.63–3.74) at 6 months (*p* < 0.001) and 3.74 g/dL (95% CI: 3.68–3.81) at 12 months (*p* = 0.009). No significant difference was observed between the PrD < 0.6 and PrD ≥ 0.6 group at baseline *p* = 0.807. Both groups exhibited similar longitudinal trajectories, characterized by an initial drop in serum albumin at 6 months followed by stabilization over the following 6 months (Figure 2a, *p* = 0.647). No significant difference in serum albumin was found between baseline and 12 months in both groups (*p* = 0.536). Furthermore, BMI did not change over time in the whole cohort with no longitudinal difference in BMI trajectories between the two diet groups (Figure 2b, 0.461).

**Figure 3:**
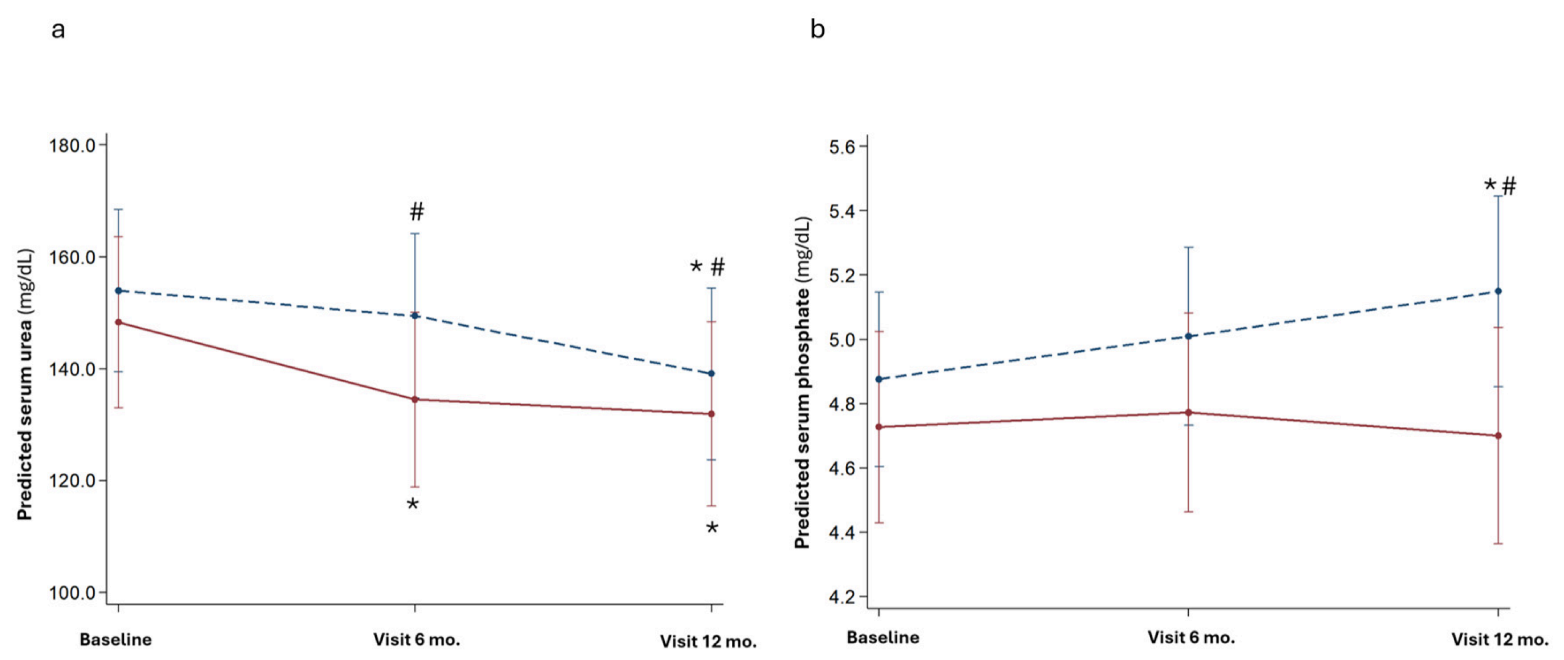
One-year changes in serum urea (panel (**a**)) and serum phosphate (panel (**b**)), ≤0.6 g/kg BW/day (solid maroon line) and >0.6 g/kg BW/day (dashed navy line). * *p* < 0.05 for intragroup comparisons; ^#^ *p* < 0.05 for intergroup comparisons.

Longitudinal trajectory of serum urea across the 12-month follow-up period does not significantly differ between two dietary groups (0.374). A significant decrease in serum urea was evident in both group and intergroup difference remained consistent throughout the 12-month follow-up (Figure 3a).

Although no difference in longitudinal trajectories of serum phosphate was not evident (*p* = 0.285), over the 12-month study period, serum phosphate levels remained stable in the PrD ≤ 0.6 group, whereas the PrD > 0.6 exhibited a significant longitudinal increase (at 12 mo.: +0.27 [95% C.I.: 0.03–0.52]; *p* = 0.030), resulting in a significant difference in serum phosphate at 12 months between PrD ≤ 0.6 and PrD > 0.6 (*p* = 0.013). There was no significant difference in use of phosphate binders between two PrD groups (0.138).

A significant group-by-time interaction was observed for serum potassium (*p* = 0.044), reflecting serum potassium control in the PrD < 0.6 whereas in the PrD > 0.6 group serum potassium increased over the study period. No difference in hemoglobin was reported over 12 months (0.504) with no difference in hb trajectories over 12 months between two groups (0.781). Notably, the longitudinal trajectory of epoetin use did not differ over time between two groups (0.670).

Body weight was similar at baseline (0.903) and was not modified by diet prescription over time (*p* = 0.477), with no significant difference at the end of follow up (*p* = 0.792). Longitudinal trajectory of systolic BP was not influenced by diet (*p* = 0.850). Accordingly, the number in anti-hypertensive drugs was similar between two groups over time (0.114). During the 12 months of follow-up, no difference in the use of renin-angiotensin system inhibitors was found in whole cohort (0.788) and the trajectories of two PRD groups were similar (0.258). The use of diuretics was significantly increased in the whole cohort (*p* = 0.005), but no difference was registered between two PRD groups (0.089).

No significant longitudinal change in serum C reactive protein was observed in shole cohort (*p* = 0.218). Longitudinal trajectory of serum C-reactive over time does not significantly differ between two dietary groups (0.193). Similarly, the trajectories of total lymphocyte count over 12 months were not different between two PrD (*p* = 0.159).

## 4. Discussion

This analysis provides novel real-world insights into the nutritional management of ESKD patients undergoing incremental PD, performing an analysis of 205 patients initiating incremental PD, aimed at comparing one-year changes in nutritional, clinical, and metabolic parameters in PrD ≤ 0.6 vs. PrD > 0.6 g/kg/day. No differences in 12-month mortality and other causes of incremental PD dropouts (switch to full-dose PD or hemodialysis, kidney transplantation) were observed between the PrD groups. We found that serum albumin levels did not significantly decrease after one year of PD regardless of the prescribed diet, and BMI remained stable in both groups over time. With comparable dialysis adequacy in both groups, defined by protocol-driven weekly total Kt/V ≥ 1.7, patients on a PrD ≤ 0.6 g/kg/day showed lower serum urea, lower serum phosphate, and lower serum potassium at the 12-month visit than those on a PrD > 0.6 g/kg/day. These findings suggest that a PrD ≤ 0.6 g/kg/day may help to control uremic toxins in patients starting incremental PD, while serum albumin levels over time remain comparable to those observed with a higher protein diet.

An interesting finding is that a significant reduction in serum albumin was observed during the first six months in the whole cohort, followed by stabilization in the subsequent six months. The observed longitudinal trajectory in serum albumin was similar in both PrD groups, suggesting that the decline may be independent of prescribed protein diet. In our study no difference in serum CRP and total lymphocytes count was observed between two PrD groups. Although this study cannot provide a pathogenic explanation of the observed serum albumin reduction, it is well known that in patients undergoing PD, the worsening in serum albumin over time is a multifactorial condition arising from the interplay of direct albumin loss into the peritoneal effluent, chronic inflammation, appetite suppression, and fluid overload [16,17].

Due to the observational and pragmatic design of the I-COPE study, decisions regarding the prescribed protein diet were left to the discretion of each participating investigator, reflecting real-world clinical practice rather than a protocol-driven approach. This methodological choice allowed us to capture how nephrologists manage nutritional prescriptions in patients initiating incremental PD. A notable finding emerging from the study is the substantial heterogeneity in dietary recommendations across centers. Such variability likely reflects the intrinsic complexity of managing patients who begin dialysis with an incremental regimen. These individuals sit in a transitional “gray zone”: they are no longer fully managed with medical care; they are not yet treated by a standard full-dose dialysis. As a result, nephrologists may differ in how they balance the goals of preserving residual kidney function, preventing malnutrition, and avoiding excessive protein restriction. This pronounced variability underscores the absence of clear, evidence-based guidance for nutritional management in this specific population and highlights the need for dedicated studies aimed at defining the optimal dietary approach for patients treated with incremental PD.

This study represents the first attempt to directly address these unmet needs in this specific patient population starting with incremental PD, suggesting a potential benefit of a lower protein diet to manage uremic complications; however, the analysis presents several limitations that prevent the drawing of any causal conclusion regarding the potential benefits of PrD ≤ 0.6 g/kg/day in patients undergoing incremental PD. Primarily, the observational design and the lack of a controlled dietary intake weaken the conclusions. Conducted as a non-profit, spontaneous study across 20 Italian PD units, nutritional prescriptions were not standardized but determined by individual nephrologists, reflecting real-world practice rather than protocol-driven management. Thus, the observational and pragmatic nature of this study is associated with a risk of “confounding by indication”, since clinicians’ decisions to prescribe a more restrictive diet (≤0.6 g/kg/day) were influenced by unmeasured factors, such as the perceived patient’s frailty, or the physician’s specific experience with certain patient profiles. Furthermore, while our models adjusted for available baseline comorbidities, we cannot rule out that patients prescribed a low-protein diet may have had a different trajectory of unmeasured potential confounders (RKF and urine volume). Although low-protein foods were routinely prescribed, adherence and quality of protein were not monitored, and actual protein intake was verified by urinary urea only at baseline; thus, some discrepancies between prescription and intake during follow up cannot be excluded. Moreover, the dietary prescriptions did not mandate a specific proportion of plant-based versus animal-based protein. Dietary counseling emphasized overall protein targets and general renal health principles, allowing for a mixed (omnivore) diet tailored to local dietary habits and patient preferences. Finally, the evaluation of sarcopenia and malnutrition relied solely on serum albumin and BMI, without complementary assessments of muscle mass or strength in the whole cohort, limiting the detection of early or subclinical nutritional impairment.

## 5. Conclusions

In conclusion, our observational analysis provides, for the first time, suggestive data on the benefits and safety of the reduction in protein in the diet of patients starting PD by incremental approach. However, the observational nature and the study limitations indicate the need for studies designed ad hoc in this setting of patients to confirm over the long term the effectiveness of this nutritional approach.

## Figures and Tables

**Table 1:** Main clinical and demographic features at baseline in the whole cohort and classified by the type of prescribed protein diet.

	Overall (N = 205)	PrD > 0.6 (N = 112)	PrD ≤ 0.6 (N = 93)	*p*
Demographics				
Age (years)	63.3 ± 14.2	64.5 ± 14.1	61.8 ± 14.3	0.168
Gender (%)	66.8%	66.1	67.7	0.800
Diabetes (%)	29.3	33.9	23.7	0.108
CVD (%)	36.1	38.4	33.3	0.453
Body Weight (kg)	70.4 ± 14.2	71.0 ± 12.6	69.8 ± 14.2	0.542
BMI (kg/m^2^)	25.5 ± 4.1	25.7 ± 4.0	25.3 ± 4.2	0.546
Systolic BP (mmHg)	135.6 ± 15.4	135.3 ± 14.9	136.0 ± 16.0	0.756
Diastolic BP (mmHg)	77.2 ± 10.8	77.4 ± 10.8	76.9 ± 10.4	0.754
eGFR at start (mL/min/1.73 m^2^)	8.5 ± 2.5	8.5 ± 2.5	8.5 ± 2.4	0.958
Serum Lab				
Urea (mg/dL)	152 ± 59	163 ± 55	139 ± 61	0.004
Sodium (mmol/L)	139 ± 4	138 ± 4	139 ± 3	0.856
Potassium (mmol/L)	4.6 ± 0.6	4.6 ± 0.6	4.5 ± 0.6	0.618
Albumin (g/dL)	3.8 ± 0.5	3.8 ± 0.4	3.9 ± 0.5	0.066
Hemoglobin (g/dL)	11.2 ± 1.2	11.2 ± 1.2	11.2 ± 1.1	0.863
Calcium (mmol/L)	8.5 ± 2.5	8.5 ± 2.5	8.5 ± 2.4	0.958
Phosphate (mg/dL)	4.9 ± 1.1	4.9 ± 1.1	4.8 ± 1.0	0.231
PTH (ng/L)	227 (116–414)	239 (123–418)	217(106–388)	0.350

CVD: cardiovascular disease; BMI: Body Mass Index; BP: Blood Pressure; GFR: Glomerular Filtration Rate.

**Table 2:** Main therapeutic features at baseline in the whole cohort and classified by the type of prescribed protein diet.

	Overall (N = 205)	PrD > 0.6 (N = 112)	PrD ≤ 0.6 (N = 93)	*p*
CAPD (%)	92.2	91.1	93.6	0.510
One PD dwell (%)	53.0	43.9	63.5	0.008
Icodextrin use (%)	64.0	61.3	67.0	0.426
Protein intake (g/day)	42.8 ± 13.6	44.8 ± 13.2	40.4 ± 13.8	0.020
N Anti-hypertensives (N)	2.2 ± 1.2	2.4 ± 1.3	2.0 ± 1.1	0.043
Furosemide (%)	68.8	72.3	64.5	0.230
Dose furosemide (mg/day)	100 (50–250)	125 (50–250)	75 (50–250)	0.814
CEIs or ARBs (%)	34.2	34.8	33.3	0.823
MRAs (%)	6.8	8.9	4.3	0.191
Calcium-antagonists (%)	65.5	63.6	67.8	0.540
β-blockers (%)	53.5	54.6	52.2	0.735
α_1_-lithics (%)	24.1	27.3	20.2	0.248
α_2_-agonists (%)	8.5	8.2	8.9	0.858
Statins (%)	47.8	47.3	48.4	0.879
Phosphate binders (%)	58.1	60.7	54.8	0.396
Calcium supplements (%)	11.4	14.4	7.7	0.129
Cholecalciferol (%)	37.1	27.7	48.4	0.002
Calcitriol (%)	34.2	31.2	37.6	0.412
Paricalcitol (%)	45.2	45.2	45.1	0.994
Epoetin (%)	58.1	64.3	50.5	0.047
Dose epoetin (units/week)	5600 (4000–8200)	6000 (4000–11,000)	4000 (2800–6000)	0.002
Potassium exchangers (%)	9.8	9.8	9.7	0.972

CAPD: continuous ambulatory peritoneal dialysis; CEI: Converting Enzyme Inhibitors; ARB: Angiotensin II Receptor Blocker; MRA: Mineralocorticoid Receptor Antagonists.

## Data Availability

The data presented in this study are available on request from the corresponding author due to privacy or ethical restrictions.

## Abbreviations

The following abbreviations are used in this manuscript:

ND-CKD	Non-dialytic chronic kidney disease
RKF	Residual kidney function
CAPD	Continuous ambulatory peritoneal dialysis
APD	Automated peritoneal dialysis
PD	Peritoneal dialysis
PrD	Protein Diet
BMI	Body Mass Index
BP	Blood Pressure
MRA	Mineralocorticoid Receptor Antagonists
CEI	Converting Enzyme Inhibitors;
ARB	Angiotensin II Receptor Blocker
CVD	Cardiovascular Disease
RRT	Renal replacement therapy
